# Predictors of job satisfaction among doctors, nurses and auxiliaries in Norwegian hospitals: relevance for micro unit culture

**DOI:** 10.1186/1478-4491-4-3

**Published:** 2006-02-17

**Authors:** Unni Krogstad, Dag Hofoss, Marijke Veenstra, Per Hjortdahl

**Affiliations:** 1 Norwegian Knowledge Centre for the Health Services, Oslo, Norway; 2 Helse Øst Centre for Health Services Research, Akershus University Hospital, Oslo, Norway; 3 Rikshospitalet, University Hospital, Oslo, Norway; 4 Department of General Practice and Community Medicine, University of Oslo, Oslo, Norway

## Abstract

**Objective:**

To explore what domains of work are important for job satisfaction among doctors, nurses and auxiliaries and to discuss differences between professional groups in the perspective of micro team culture.

**Design:**

Cross-sectional survey data from hospital staff working clinically at inpatient hospital wards in Norway in 2000.

**Measures:**

Linear regression models predicting job satisfaction for the three professions were compared. First, five domains of hospital work were examined for general job satisfaction. Based on the result of the first regression, five items concerning local leadership were explored in a second regression.

**Results:**

A total of 1814 doctors, nurses and auxiliaries working at 11 Norwegian hospitals responded (overall response rate: 65%). The only domain of work that significantly predicted high job satisfaction important for all groups was positive evaluation of local leadership. Both steps of analyses suggested that professional development is most important for doctors. For registered nurses, experiencing support and feedback from the nearest superior was the main explanatory variable for job satisfaction. Job satisfaction of auxiliaries was equally predicted by professional development and local leadership. The results are discussed and interpreted as reflections of cultural values, loyalties and motivation.

**Conclusion:**

The professional values of medicine, the organizational and holistic skills of nurses and the practical experience of auxiliaries should all be valued in the building of interdependent micro teams.

## Background

In recent years the link between hospital quality, organizational culture and management has received increasing attention in the United States of America [1-4] as well as in Europe [5-7]. Reflecting the restructuring programmes of health services organizations at the macro level, most of the literature on hospital organization and management has related to the level of the institution or above. In the United States, however, there has been a movement towards focusing on the local levels of organizations, with the argument that no system can be better than the microsystems of which it is composed [8-12]. Research on the function and culture of microsystems is scarce, however.

Microsystems in health service, as defined by Nelson et al. [11], are small teams working together on a regular basis to serve the needs of a discrete subpopulation of patients. Microsystems by this definition include patients, clinicians, processes and recurring patterns and are described as complex adaptive systems. This definition is a good fit with hospital wards, which in a Norwegian setting constitute the smallest permanent organizational units. The professional groups working together with the patients on a daily basis in a ward are doctors, nurses and auxiliaries. There is however, substantial variation between hospital wards when it comes to work organization, cooperation and working climate. A series of articles in the *Joint Commission Journal on Quality and Safety *[8-16] discusses the characteristics of units that succeed in motivating staff for quality improvement and high performance.

High-performance units, measured by medical records as well as financial reviews, were assessed and nine success characteristics identified: leadership of microsystem, culture of microsystem, interdependence of care team, macro-organizational support of microsystem, patient focus, staff focus, information and information technology, process improvement and performance pattern [11]. The positive effects suggested were the ability to provide superior, cost-effective care and at the same time to create a positive and attractive working environment.

The present study focuses on three of the identified success criteria: leadership of microsystem, interdependence of care team and differences in professional cultures in a setting of hospital wards. A strong relationship between quality of care and leadership has been suggested by several authors [17,18]. Other studies have shown positive relationships between job satisfaction and quality of patient care [19,20]. In a microsystem such as a hospital ward, managing the diversity of professional cultures [21] might be a key factor for establishing interdependent ward teams as well as increasing satisfaction - both of them means to improved quality of care.

The concept of culture has been disputed for years within social anthropology [22,23]. A very pragmatic perspective is that culture is the competence of "how we do things around here" [24]. This perspective suggests that culture manifests itself and may be studied through actions, shared beliefs and values. For further development of this perspective, Schein's [25] analytical framework of culture may be useful. Schein identifies three different but interdependent levels of culture: the first level he calls *artifacts*. This is what we see: actions and representations, visible for observers and openly communicated. At the second level he places *beliefs and values *that include evaluations and judgements. These are not visible but mostly accessible by questionnaires or other methods. The third level of culture, which he calls *assumptions*, are the deep formatting of mind - values so internalized in individuals and organizations that they are taken for granted.

To get knowledge about the different levels in Schein's model will require different research methods. Artifacts and values may be directly observed or may be registered as variables in surveys. Deep beliefs and assumptions, however, would ideally require longitudinal ethnographic observations to provide valid knowledge.

Culture is produced through shared challenges, discussions and communication over a period of time [26].

Interdependence of the care team is described by Nelson et al. as a culture characterized by trust, collaboration, willingness to help each other, appreciation of complementary roles and a recognition that all contribute to a shared purpose [11]. Depending on the size of the microsystem, this kind of trust may start developing in even smaller groups, such as groups working on the same shifts or working with subgroups of patients within a ward. In Norway such teams typically consist of three professions: doctors, nurses and auxiliary nurses. To distinguish between different levels within the micro system, a ward will be called "micro unit" and smaller working teams "micro teams". Whether such micro teams are structurally organized or emerge more ad hoc according to tasks and challenges, a culture characterized by trust and collaboration is a crucial matter for which leaders are responsible [6,18,27]. Research on what cultural traits within the professions may facilitate or hamper the establishment of teams characterized by trust and collaboration is scarce.

We suggest that variables of artefacts, like evaluation of competence, organization of work and leadership, may provide a reflection of deeper assumptions. These reflections may differ between professions. In this article we use data on job satisfaction of three professional groups. The aim of the article is to interpret values and assumptions by analysing which domains of work predict job satisfaction of doctors, nurses and auxiliaries, and to discuss the relevance of these assumptions for micro level culture.

## Methods

### Data collection

A postal survey including all types of personnel at 11 acute somatic hospitals across Norway was performed in 2000. The participating hospitals represented two out of the five university hospitals in Norway, three out of the 12 county hospitals and six local hospitals in the country. All five health regions in Norway were represented.

Nurses (three years' college education) and auxiliary nurses (18 months' vocational training) are employed at ward level in Norway. Their immediate leader is a head nurse. Doctors are employed at department level but they usually have their daily work in one particular ward together with the nursing staff. The immediate leader for doctors is most often a specialist or consultant.

For the purpose of this article we included in the sample all doctors, nurses and auxiliary nurses working more than 50 % of full time. Names and addresses were obtained through the personnel administration systems of the participating hospitals. Non-respondents received one reminder after two to three weeks. Due to the aims of anonymity of the study, our only information about non-respondents retained after the reminder procedure was profession and hospital.

### The questionnaire

Work experiences were measured by the Work Research and Quality Improvement questionnaire (WORQUA). As one major interest was to investigate organizational issues of hospital work, which is more multifaceted and not easy to transfer from one health care system to another, this questionnaire combined items and scales from established job-satisfaction questionnaires [28-30] with additional items designed to map the Nordic model of hospital work organization [31].

After a large-scale study in 1998 that included 15 hospitals, the questionnaire was modified to strengthen the dimensions of work organization and professional development before the 2000 study [32]. All items were recoded so that higher numerical values represented higher levels of satisfaction. For this article, five indices of work experiences were constructed on the basis of factor analysis and theoretical considerations. Items and attributes of each scale are listed in Table 4.

### Statistical analysis

According to our suggestion that cultural values and assumptions may be interpreted from what constitutes job satisfaction, we set up a multiple linear regression model to analyse the relative contributions of five indices on the dependent variable: "All things considered, how satisfied are you with your job?" The five indices were top management, local leadership, competence, work organization and professional development. The dependent variable was measured on a five-point Likert scale ranging from "very dissatisfied" to "very satisfied". Self-reported age was included in the regression as the controlling variable because earlier analyses show that increasing age significantly contributes to job satisfaction [32]. Sex is not included as a correcting variable, because the fact that almost all nurses were female and doctors mostly male is a characteristic of the sample that should not be "corrected". Separate regression models were specified for each of the three professions.

We expected the three professions to differ in their explanatory models. If the three groups did not differ on the level of indices, we wanted to explore whether there were differences at a more detailed level, by doing a second regression using single items as explanatory variables. Again, separate analyses for each professional group were conducted. Explanatory models are summarized by Adjusted R-squares. Statistical analyses were performed using SPSS 11.5 software for Windows.

## Results

### Respondents

Of the 2790 nurses, doctors and auxiliaries receiving a questionnaire, 1814 answered after one reminder. The response rates for nursing staff was 66% and for physicians 62%. The number in each profession in our study accounted for approximately 5% of the nurses, doctors and, auxiliaries employed in Norwegian acute somatic hospitals in 2000. The nurses in the study were fairly equally distributed among hospital types: university hospitals (33%), county hospitals (34%) and local hospitals (34%), while the other professions differed between 27% and 45% (Table 1). Response rates differed from 59% to 68% between hospital types, with county hospitals representing the highest and university hospitals the lowest. Further characteristics of responders are described in Table 1.

**Table 1:** Description of respondents (N = 1814)

	**Doctors N = 358**		**Nurses N = 1066**		**Auxiliaries N = 390**	
	**Doctors N = 358**		**Nurses N = 1066**		**Auxiliaries N = 390**	
**Sex**						
Male	254	73%	72	7%	12	3%
Female	92	27%	976	93%	361	97%
**Age**						
<30	34	10%	360	34%	43	11%
30–39	131	37%	340	32%	63	16%
40–49	88	25%	240	23%	131	34%
50–59	76	22%	90	9%	125	33%
60+	23	6%	23	2%	22	6%
**Hospital level**						
University hospitals	105	29%	347	33%	107	27%
County hospitals	160	45%	358	34%	122	31%
Local hospitals	93	26%	361	34%	161	41%
**Department**						
Acute/special	6	2%	100	9%	0	
Surgery	150	42%	386	36%	171	44%
Medical	193	54%	545	51%	184	47%
Rehabilitation	9	2%	35	3%	35	9%

### Statistical analysis

The five indices used in the first regression models showed acceptable internal consistency, with Cronbach coefficient alphas above the critical value of 0.70 for all indices (Table 4). The dependent variable was checked for satisfactory distribution of standardized residuals.

### First regression models

Satisfaction with their local leader was the most important explanatory variable for nurses' job satisfaction. Positive evaluation of top management as well as work organization also significantly predicted job satisfaction (Table 2). Adjusted R^2 ^for this model was 0.29.

**Table 2:** Effects of index predictors on job satisfaction for nurses, auxiliaries and doctors (Beta, significance of difference, B and 95% CI)

	**Beta**	***P*value**	**B**	**95% CI for B**
**Nurses N = 1066**				
(Constant)		= .00	1.86	(1.51–2.21)
Age	0.03	0.33	0.02	(-0.02–0.07)
Top management	0.18	<0.001	0.18	(0.10–0.26)
Local leadership	0.23	<0.001	0.19	(0.12–0.26)
Competence	0.00	0.90	0.00	(-0.04–0.04)
Work organization	0.19	<0.001	0.07	(0.04–0.11)
Professional development	0.06	0.11	0.06	(-0.01–0.12)
Adjusted R^2 ^= 0.293				
**Auxiliaries N = 390**				
(Constant)		0.00	1.46	(0.84–2.08)
Age	0.05	0.39	0.03	(-0.04–0.11)
Top management	0.10	0.21	0.10	(-0.06–0.25)
Local leadership	0.20	0.01	0.18	(0.05–0.32)
Competence	0.14	0.03	0.08	(0.01–0.14)
Work organization	0.03	0.70	0.01	(-0.04–0.06)
Professional development	0.21	0.002	0.18	(0.07–0.30)
Adjusted R^2 ^= 0.251				
**Doctors N = 358**				
(Constrant)		0.00	1.26	(0.61–1.91)
Age	-0.13	0.01	-0.10	(-0.18– -0.02)
Top management	0.05	0.47	0.05	(-0.08–0.17)
Local leadership	0.22	0.00	0.23	(0.10–0.36)
Competence	0.07	0.22	0.05	(-0.03–0.12)
Work organization	0.12	0.09	0.05	(-0.01–0.11)
Professional development	0.37	<0.001	0.34	(0.23–0.45)
Adjusted R^2 ^= 0.398				

For auxiliaries the prime predictor for job satisfaction was working in a culture of professional development. Competence and local leadership also were important for their job satisfaction (Adjusted R^2 ^= 0.25).

The strongest predictor of high job satisfaction for doctors was to work in a culture of professional development. Job satisfaction also increased with positive perceptions of the local leader. Age showed a negative effect on job satisfactions for doctors. Adjusted R^2 ^for the model was 0.37. The only index that was a significant predictor for all three groups was the domain of local leadership.

### Second regression models

A positive evaluation of the local leader was shown to be a significant predictor of job satisfaction for all groups. By exploring this domain alone in more detail, we found that the different professions emphasized different aspects of local leadership. Experiencing support and encouragement from the immediate superior was most important for nurses' job satisfaction (Table 3). Professional feedback and the feeling that the nearest leader knows the work situation were also significant predictors. For auxiliaries the superiors' knowing the work situation was the most important predictor of job satisfaction, but encouragement and support were also important. The models explained 20% for nurses and 21 % for auxiliaries, as measured by Adjusted R^2^. The immediate leaders' knowing the work situation and providing professional feedback were the most important explanatory variables for doctors' job satisfaction (Adjusted R^2 ^= 0.18).

**Table 3:** Effects of single item predictors on job satisfaction for nurses, auxiliaries and doctors (Beta, significance of difference, B and 95% CI)

	**Beta**	***P*value**	**B**	**95% CI for B**
**Nurses N = 1066**				
(Constant)				
11-5 My superior knows my job situation*	0.18	<0.001	0.14	(0.09–0.20)
14-1 My superior encourages and supports me*	0.21	<0001	0.15	(0.08–0.22)
14-2 My superior provides feedback on my work*	0.10	0.02	0.06	(0.01–0.12)
14-5 My superior always speaks clearly*	0.04	0.35	0.03	(-0.03–0.08)
14-6 My superior is an important inspiration*	0.00	0.95	0.00	(-0.06–0.06)
Adjusted R^2 ^= 0.203				
**Auxiliaries N = 390**				
(Constant)		0.00	2.37	(2.02–2.72)
11-5 My superior knows my job situation	0.25	0.00	0.20	(0.11–0.29)
14-1 My superior encourages and supports me	0.17	0.02	0.12	(0.02–0.23)
14-2 My superior provides feedback on my work	0.11	0.10	0.07	(-0.01–0.16)
14-5 My superior always speaks clearly	-0.08	0.28	-0.06	(-0.16–0.05)
14-6 My superior is an important inspiration	0.12	0.09	0.08	(-0.01–0.17)
Adjusted R^2 ^= 0.214				
**Doctors N = 358**				
(Constant)		0.00	2.20	(1.81–2.58)
11-5 My superior knows my job situation	0.15	0.02	0.13	(0.02–0.24)
14-1 My superior encourages and supports me	0.04	0.59	0.04	(-0.09–0.16)
14-2 My superior provides feedback on my work	0.16	0.03	0.12	(0.01–0.24)
14-5 My superior always speaks clearly	0.05	0.40	0.04	(-0.05–0.13)
14-6 My superior is an important inspiration	0.14	0.05	0.11	(0.00–0.22)
Adjusted R^2 ^= 0.184				

*All items are shortened in the table. For the full phrase, please see Table 4.

**Table 4:** Indices and questions

**Index 1. Top management **(Cronbach's alpha = 0.76; 4 questions, scale 1–5)
**Index 1. Top management **(Cronbach's alpha = 0.76; 4 questions, scale 1–5)
11-3: The department management knows the job situation in the wards.
11-4: In my department, the management's priorities are good.
11-6: The hospital top management really makes an effort to keep the staff.
**Index 2. Local leadership **(Cronbach's alpha = 0.85; 5 questions, scale 1–5)
11-5: My immediate superior knows my job situation well.
14-1: I get the encouragement and support I need from my immediate superior.
14-2: My immediate superior provides feedback so that I know whether I'm doing a good job.
14-5: My immediate superior always speaks clearly.
14-6: My immediate superior is an important inspiration.
**Index 3. Competence **(Cronbach's alpha = 0.77; 3 questions, scale 1–10)
15-5: How would you rate the competence of the nurses in this ward?
15-6: How would you rate the competence of the doctors in this ward?
15-7: How would you rate the competence of the auxiliaries in this ward?
**Index 4. Work organization **(Cronbach's alpha = 0.82; 3 questions, scale 1–10)
16-1: Expectations from superiors are clear.
16-2: Tasks are clearly defined.
16-6: The work organization is good.
**Index 5. Professional development **(Cronbach's alpha = 0.74; 2 questions, scale 1–5)
25-1: The work environment is characterized by professional development.
25-4: The hospital provides good opportunities for professional development.

## Discussion

A surgical ward of about 24 beds in a Norwegian hospital may have two to four consultants (specialists) permanently attached to the ward, in addition to five to eight doctors in educational positions working at the ward for six to 12 months. All doctors participate in a rota duty within their specialty that includes emergency calls and ambulatory duties. About 20 to 25 nurses and four to six auxiliaries working solely in the ward constitute the nursing staff.

Within a ward, working teams consisting of doctors, nurses and auxiliaries care for smaller groups of patients. Shifts and different working hours make such teams unstable, with alternating individuals. Establishing good working teams within the micro unit is a vital challenge for local leaders; managing the cultural diversity of professions is a central part of that challenge [21,33]. To secure safety as well as flexibility in the work process, development of shared values, shared aims and a common language among the team members is vital. That the members of the three professional groups differed in their opinions as to what constituted the most important elements for their job satisfaction implies that leaders should take these differences into account in their efforts for motivation and team building.

### Interpreting nurses' regression models

The predictors of job satisfaction of nurses included organizational and professional as well as personal dimensions. The first regression showed the importance of leadership and organization. Focusing on local leadership, the importance of being encouraged and supported by the nearest leader points to a need to be seen and appreciated. Aiken and others found that nurses experience frustration and burnout because of lack of control over work conditions that determine the job for which they are responsible [34,35]. Other studies have shown that nurses felt they were not treated as clinicians or peers by doctors and hospital managers but as assistants, at risk of being replaced by less-qualified personnel who cost less to employ [20,36].

Studies of nurse burnout and magnet hospitals in the United States concluded that professional development, cooperation with medical staff and managerial support were highly important for nurses [37,38]. In this study the influence of good opportunities for professional development on nurses' job satisfaction was not significant, seen in combination with other factors. One interpretation may be that competence and professional development are closely intertwined with the local organization of work.

The significant predictors of the nurses' explanatory model included the local leader's knowing the work situation and providing support and feedback. The head nurse is, among other things, responsible for the local education and fostering of new nurses. Professional development might therefore be seen as taken care of through the local leader.

Work organization (Index 4) was also an important predictor for nurses' job satisfaction. This is in line with findings that cohesive working relationships, cooperation with medical staff and appropriateness of the system of nursing was important for nurse job satisfaction in the United Kingdom [39].

Nurses seem to be concerned about the vertical as well as horizontal organizational coherence in their work, which may reflect the multidimensionality of nurses' work. Because nurses have a coordinating role, the responsibility for shuttling between professional, organizational and relational tasks makes them utterly aware of organizational gaps and inconsistencies. Nurses inhabit an organizational position from which they overlook the local system's impact on professional competence as well as cooperation and workflow. Their system competence is an important asset in the micro team and should be used by managers.

### Interpreting auxiliaries' regression models

Auxiliaries are the group with the least professional authority. In our data 98% of the auxiliaries were female, 75% were above 40 years of age and 60% worked part-time. They are however, also the most stable group in hospital wards, thus representing long experience and considerable informal knowledge about their local patient groups as well as the hospital organization.

It may seem surprising that professional development and working in a unit with high competence is important for their job satisfaction, while professional feedback is not a significant predictor. One interpretation is that auxiliaries draw their professional identity and loyalty more from the collective of the micro unit than from their own professional group. Having little chance of formal promotion, their prospects for professional acknowledgement and respect lie in building informal competence and local reputation. For the micro team these kinds of local skills and loyalties are vital. In building an interdependent care team, a leader's task should be to develop the collective aspect of the ward's total situational competence and hence to make the other professional groups acknowledge this resource as an asset.

### Interpreting doctors' regression models

The strongest predictor of doctors' job satisfaction was working in a culture of professional development. Having a leader who knows the work situation and gives feedback on the work was also seen as important. The nearest superior for doctors is usually a consultant or a clinical manager. Knowing the work situation means understanding the difficulty and variation of clinical assessment and having the capacity to give professional feedback. The most striking cultural trait emerging from the doctors' results is the noticeable importance of the profession. The doctors' explanatory model reflects loyalties, authorities and motivation linked to the medical profession only. Studying trends in the doctor – manager relationships in the United Kingdom, Davies et al. support the picture of doctors' loyalty: Doctors reproduce a professional and individualistic authority [40,41]. In the perspective of an interdependent ward culture, the doctors' knowledge and professional responsibility are valuable contributions. The challenge for leaders could be to extend this professional loyalty and responsibility to the multiprofessional team as an investment in the collective assets of the ward.

### Implications for quality improvement

A common strategy for bridging the gap between managerial and clinical rationalities in hospitals has been to train doctors and nurses in managerial theory and methods. The undesirable side effects of losing clinical responsibility and caring morale integrated in the clinical cultures have hardly been discussed [42,43]. A supplementary strategy would be to teach managers to recognize clinical values and cultures and thus to use the strength of each professional group. The respondents in our study pointed clearly to the importance of having leaders know and understand the different working situations. We also suggest that leaders pay attention to the values of the different professions of the micro team. This supports the point in what Firth-Cozens calls a fundamental conflict of leadership for quality: the necessity for hospital leaders to get close to the patient and staff experience [6].

By exploring what predicted their job satisfaction, we have shed some light upon the values and the underlying assumptions of the different clinical professions at ward level. Such insights may be used as motivation through differing strategies tailored for each profession. Some crucial points may be to strengthen confidence and motivation at the local level by rewarding doctors for their professionalism but also urging them to share their knowledge and to expand their professional responsibility to the micro team as a whole. Nurses and auxiliaries could be used systematically as informants on the functioning of the micro- and meso-level of the hospital infrastructure. The coordinating role of nurses and the stability and local experience of auxiliaries give them an organizational overview that obviously is useful in quality improvement strategies.

### Strengths and limitations

The response rate of the study was < 70%, which may be considered low. As our sample was made unidentifiable after the inclusion procedure, our knowledge about non-respondents is limited. All regions and hospital sizes in Norway were represented, however, with all three professions in the study; there is no reason to believe that our sample was in any way biased.

The use of identical questions to different professions risks differing interpretations from the various groups. This may represent a validity problem, but on the other hand it also allows direct comparison between professional groups. The fact that gender still is closely linked to professions represents some interpretation problems. We cannot say that the values described are rooted in professions and not in gender. On a group level, though, we may state that professionalism is a cultural trait of hospital physicians.

Hospital staff work within hospitals, departments and wards and thus share experiences and attitudes with their local colleagues. This would lead to underestimated standard errors and thus an inflated type I error rate. As we were not able to link all professional groups to ward levels of work, which is the most likely level of clustering, multilevel analysis could not be performed.

To interpret a survey of work experiences as cultural information requires caution. We do not recommend the method for cultural studies as such. We do, however, defend the possibility of searching such material to identify characteristics reflecting cultural main vectors at a relatively basic level. In a setting of managing quality improvement, this level might be sufficient for practical purposes.

## Competing interests

The author(s) declare that they have no competing interests.

## Authors' contributions

Unni Krogstad was responsible for the survey and the design of the paper; she wrote the first draft of the article. Dag Hofoss and Marijke Veenstra participated in the statistical analysis and revisions of the article. Per Hjortdahl contributed ideas throughout the process and to revisions of the paper.
